# DynTransNet: Dynamic Transformer Network with multi-scale attention for liver cancer segmentation

**DOI:** 10.3389/fonc.2025.1569083

**Published:** 2025-06-19

**Authors:** Siming Zheng, A. S. M. Sharifuzzaman Sagar, Yu Chen, Zehao Yu, Shi Ying, Yongyi Zeng

**Affiliations:** ^1^ The First Affiliated Hospital of Fujian Medical University, Fuzhou, China; ^2^ Department of Hepatopancreatobiliary Surgery, First Hospital of Ningbo University, Ningbo, China; ^3^ Department of Artificial and Robotics, Sejong University, Seoul, Republic of Korea; ^4^ AI Lab, MetaSyntec Co., LTD, George Town, Cayman Islands; ^5^ Department of Engineering Technology, Ningbo Wedge Medical Technology Co., LTD, Ningbo, China; ^6^ Department of Hepatopancreatobiliary Surgery, Mengchao Hepatobiliary Hospital of Fujian Medical University, Fuzhou, China

**Keywords:** liver cancer, segmentation, deep learning, hepatocellular carcinoma, liver tumor

## Abstract

**Introduction:**

Hepatocellular carcinoma (HCC), a predominant subtype of liver cancer, remains Q7 a major contributor to global cancer mortality. Accurate delineation of liver tumors in CT and MRI scans is critical for treatment planning and clinical decision-making. However, manual segmentation is time-consuming, errorprone, and inconsistent, necessitating reliable automated approaches.

**Methods:**

This study presents a novel U-shaped segmentation framework inspired by U-Net, designed to enhance accuracy and robustness. The encoder incorporates Dynamic Multi-Head Self-Attention (D-MSA) to capture both global and local spatial dependencies, while the decoder uses skip connections to preserve spatial detail. Additionally, a Feature Mix Module (FM-M) blends multiscale features, and a Residual Module (RM) refines feature representations and stabilizes training. The proposed framework addresses key challenges such as boundary precision, complex structural relationships, and dataset imbalance.

**Results:**

Experimental results demonstrate superior segmentation performance, achieving a mean Dice score of 86.12 on the ATLAS dataset and 93.12 on the LiTS dataset.

**Discussion:**

The proposed method offers a robust, efficient tool for liver tumor segmentation and holds strong potential to streamline diagnostic workflows and improve automated medical image analysis in clinical practice.

## 1 Introduction

Hepatocellular carcinoma (HCC), the predominant form of liver cancer, ranks as the sixth most common cancer and the third leading cause of cancer-related deaths globally, accounting for over 800,000 deaths each year (1–3). The high mortality rate is largely attributed to late diagnosis and the aggressive progression of liver tumors. In clinical practice, early detection and precise delineation of liver tumors are essential for improving patient survival rates, enabling timely interventions such as surgical resection, radiofrequency ablation, or targeted therapy. Accurate tumor segmentation in imaging modalities like computed tomography (CT) and magnetic resonance imaging (MRI) plays a critical role in guiding treatment planning, monitoring therapeutic response, and reducing inter-observer variability among radiologists. However, manual segmentation of liver tumors is labor-intensive, time-consuming, and prone to human error. To address these challenges, there is a growing demand for automated segmentation frameworks that provide consistent, efficient, and accurate tumor identification.

Automated segmentation involves the use of computational algorithms to extract meaningful regions—such as organs, tissues, or tumors—from medical images without manual intervention. This is typically achieved through deep learning-based feature extraction and boundary delineation. Several researchers have explored such approaches for liver cancer segmentation. Ayalew et al. presented a modified U-Net architecture tailored for abdominal CT images, addressing class imbalance challenges (4). Zang et al. used pulse-coupled neural network preprocessing combined with SE-ResNet to effectively denoise and segment liver tumors from CT and MRI scans (5). Amin et al. proposed a three-phase strategy involving synthetic data generation using GANs, YOLOv3-based tumor localization, and Deeplabv3 for precise segmentation (6). While these approaches demonstrate notable progress, critical challenges remain.

Liver tumor segmentation is complicated by variations in tumor shape, size, and contrast, as well as the presence of ambiguous boundaries and overlapping tissue structures. Even with advancements in convolutional neural networks (CNNs) and transformer-based models, existing methods often fail to capture complex structural relationships and achieve reliable boundary precision. These limitations are further compounded by class imbalance in medical datasets, which can bias the model toward dominant structures.

To address these issues, we propose a novel segmentation framework based on a U-shaped architecture inspired by U-Net. Our model integrates a Dynamic Multi-Head Self-Attention (D-MSA) mechanism in the encoder to capture both global and local spatial dependencies. The decoder incorporates skip connections to retain high-resolution spatial features, while a Feature Mix Module (FM-M) enables multi-scale feature fusion and a Residual Module (RM) supports stable training through efficient feature refinement. Together, these components enable accurate segmentation of liver tumors, effectively addressing boundary precision, structural integration, and data imbalance. The proposed framework offers a clinically relevant, robust tool for medical image analysis.

## 2 Related works

Automated segmentation of liver tumors has undergone remarkable progress, driven by advancements in both traditional machine learning and deep learning techniques. Early architectures like U-Net introduced an encoder-decoder structure that set a benchmark for pixel-level segmentation in medical imaging. Its successors, such as U-Net++ and 3D U-Net, enhanced feature aggregation and volumetric segmentation by incorporating nested and dense skip connections, effectively addressing spatial correlations in CT and MRI scans. These foundational models laid the groundwork for subsequent innovations that integrate attention mechanisms and hybrid approaches to further improve segmentation performance.

Recent developments have emphasized the integration of advanced architectures and attention mechanisms. Lal et al. developed a deep learning framework for nuclei segmentation in histopathological liver cancer images, integrating residual blocks to enhance high-level feature extraction and attention based decoder modules for precise spatial localization. Their architecture demonstrated state-of-the-art performance on benchmark datasets, including KMC Liver and Kumar, outperforming existing methods in segmentation accuracy (7). Similarly, Suganeshwari et al. developed EN-DENet, an encoder decoder network for liver tumor segmentation, reporting Dice scores of 85.94% on the LITS dataset and 84.81% on the 3DIRCADb01 dataset (8). Zhang et al. developed a 3D CNN architecture incorporating multi-scale convolutional layers, residual pathways, and channel-spatial attention modules to enhance hierarchical feature learning. Their framework demonstrated robust performance, yielding Dice coefficients of 76.5% and 72.96% on the LiTS and 3DIRCADb01 datasets, respectively, outperforming prior approaches in liver lesion segmentation tasks (9). Lei et al. enhanced liver and tumor segmentation using a deformable encoder-decoder network with Ladder-ASPP modules, achieving a Dice score of 76.7% on the LiTS dataset (10).

Lambert et al. developed a novel segmentation framework leveraging anisotropic hybrid networks to delineate liver and tumor regions in contrast-enhanced MRI (CE-MRI) data. Their methodology combined dual-binary pipelines (multi-class and dual-binary architectures) integrated with uncertainty quantification mechanisms to improve model transparency and computational efficiency, outperforming conventional approaches in both accuracy and reliability (11). Patel et al. focused on robust liver segmentation in T1-weighted MRI, leveraging architectures like Swin UNETR, nnUNet, and PocketNet trained on a multi-institutional dataset of 819 images, achieving Dice scores above 0.9. Their study highlights the impact of ensemble datasets and tailored models for addressing variations in imaging protocols and disease etiologies (12).

Transfer learning has also been explored to improve segmentation. Ye et al. evaluated pre-trained STUNet models for volumetric medical image segmentation, demonstrating strong modality transfer capabilities and superior performance on diverse datasets, particularly in cases of limited data (13). Li et al. presented MFHARFNet, a multi-branch hybrid attention and adaptive receptive field network, redesigning skip connections and integrating multi-scale attention modules to achieve high performance on datasets such as ATLAS, LiTS, and BraTS2019 (14).

In undersampled MRI data, Duan et al. proposed TJLD-Net, a dual-domain network combining CNNs and Transformers in an end-to-end joint learning approach for MRI reconstruction and segmentation. Their method effectively addressed the challenges of low-quality imaging (15). Dai et al. introduced SoSegFormer, a cross-scale feature correlated network for small object segmentation, demonstrating effectiveness in datasets like ATLAS and PolypGen through cross-scale feature aggregation and vision transformers (16). Finally, Qin and Li proposed S MedNeXt, a hybrid ConvNeXtTransformer model for 3D reconstruction of liver and tumors, achieving high efficiency and accuracy with a Dice score of 0.934 on the ATLAS dataset (17).

Despite these advancements, many existing methods continue to face challenges in achieving precise boundary delineation, particularly for tumors with irregular shapes or overlapping regions. Additionally, the inability to fully capture complex structural relationships within the liver remains a significant limitation. These issues are further exacerbated by imbalanced datasets, which can bias models to focus on prominent features while neglecting smaller or less distinct regions. Addressing these limitations is crucial for developing more robust and accurate segmentation frameworks, motivating the design of our proposed method.

## 3 Materials and methods

**Figure 1:**
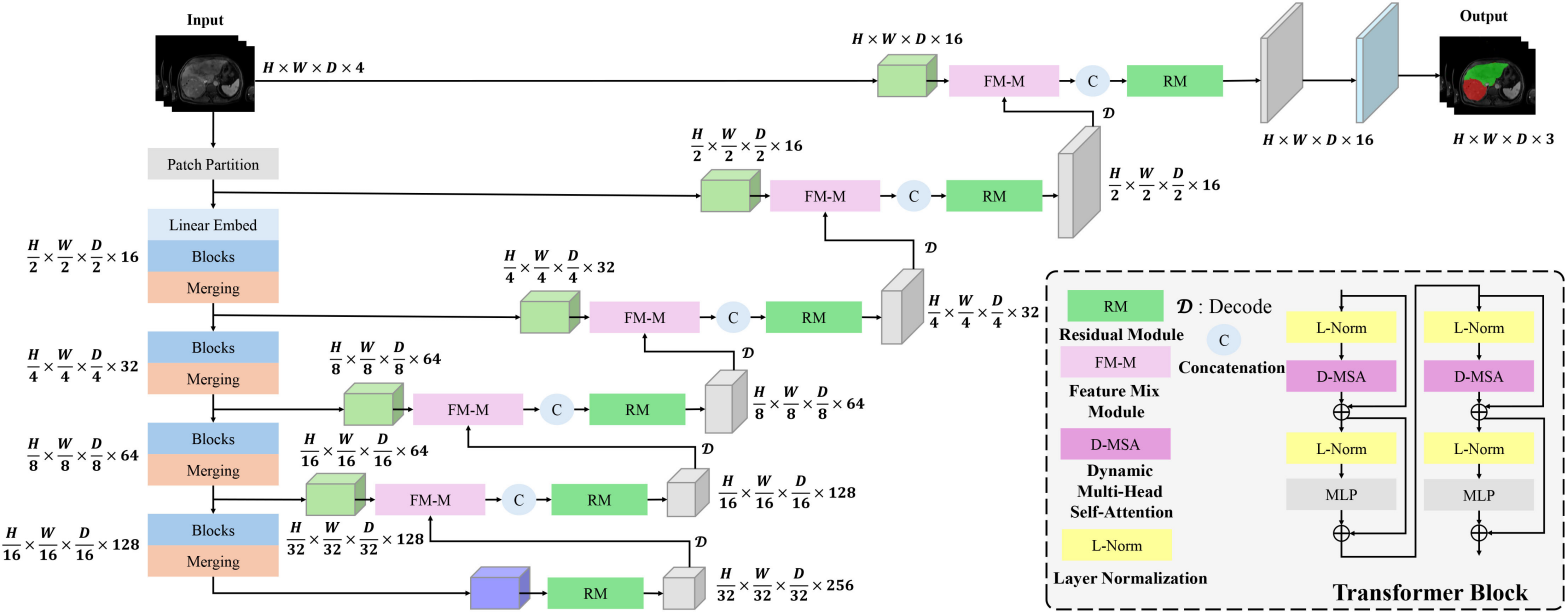
DynTransNet’s architecture comprises three main components: a transformer block, a FM-M, and a RM.

**Figure 1** illustrates the overall architecture of the proposed Dynamic Transformer Network (DynTransNet), which is based on the UNETR framework and follows an encoder–decoder structure. The encoder is responsible for extracting and compressing multi-scale features from the input images. It begins with patch partitioning and linear embedding, followed by multiple Transformer Blocks that incorporate D-MSA to effectively capture both global and local spatial dependencies. As shown in **Figure 1**, the output tokens from each encoder stage are progressively downsampled through patch merging operations, which are clearly depicted by the narrowing feature pathways. The decoder, on the other hand, reconstructs high-resolution segmentation masks by progressively upsampling these compressed representations. A key novelty of our architecture is the integration of the FM-M and RM at each decoding stage. The FM-M fuses features from the skip-connected encoder path and the upsampled decoder features via an attention-guided mechanism. The output of this fusion is then passed through the RM, which refines the combined feature maps using convolutional blocks and residual learning to ensure stable training and improved boundary precision. These modules are repeated at each stage of the decoder and are visually highlighted in the figure to illustrate their flow and interconnections.

### 3.1 Encoder

In our proposed architecture, the encoder operates on an input tensor *X* ∈ ℝ*^H^* ^×^ *^W^* ^×^ *^D^* ^×^ *^S^*, where *H*, *W*, and *D* denote the spatial dimensions, and *S* indicates the number of input channels. The encoder leverages a transformer-based architecture with D-MSA to capture both global and local spatial dependencies dynamically and efficiently. The input tensor X is first partitioned into non-overlapping 3D patches of size *H*_0_ × *W*_0_ × *D*_0_ to create a sequence of tokens with dimensions:


$$\left[\frac{H}{H_{0}}]\times [\frac{W}{W_{0}}]\times [\frac{D}{D_{0}}\right]$$

Each token is subsequently mapped into a learnable C-dimensional embedding space through a linear projection.


$$E=X^{\prime }W_{e}+b_{e}$$

where $X^{\prime }$ is the flattened representation of the tokens, $W_{e}\in {\mathbb{R}}^{H_{0}W_{0}D_{0}\times C}$ is the projection weight matrix, and $b_{e}\in {\mathbb{R}}^{C}$ is the bias term.

The core of the encoder consists of a series of transformer blocks, each equipped with DMSA to effectively model multi-scale spatial interactions. At each layer l, the input $X_{l}\in {\mathbb{R}}^{N\times C}$ , where $N=\frac{H}{H_{0}}\times \frac{W}{W_{0}}\times \frac{D}{D_{0}}$ . The detailed description of DMSA is given below,

#### 3.1.1 Dynamic Multi-Head Self-Attention

We introduce D-MSA to make the attention mechanism more adaptive to diverse image contexts. This mechanism extends the standard self-attention by incorporating a dynamic bias term that modulates attention weights based on the input features themselves. Input features are first projected into query, key, and value vectors that help model relationships between spatial positions. Unlike fixed attention formulations, D-MSA introduces a learnable, input-dependent bias that adjusts how strongly the model attends to different regions. This dynamic bias is computed from the input using a ReLU-activated linear transformation and added to the attention score before softmax normalization. As a result, the model can selectively emphasize more relevant spatial features based on the current context. By concatenating the outputs from multiple attention heads and projecting them back into the feature space, D-MSA enables richer and more flexible representation learning, effectively capturing both global context and fine-grained local details, which is critical for segmenting complex anatomical structures like liver tumors.

Within each attention head, input features are independently mapped via linear transformations to specialized query (Q), key (K), and value (V) vectors to enable distinct representation learning for contextual alignment as follows,


$$Q_{h}=X_{l}W_{q}^{h},K_{h}=X_{l}W_{k}^{h},V_{h}=X_{l}W_{v}^{h}$$

where $W_{q}^{h},W_{k}^{h},W_{v}^{h}\in {\mathbb{R}}^{C\times d_{h}}$ are learnable parameters, and $d_{h}=\frac{C}{H}$ is the dimensionality of each head. The scaled dot-product attention with dynamic bias is then computed as follows,


$$A_{h}=\text{Softmax }\left(\frac{Q_{h}K_{h}^{⊤}}{\sqrt{d_{h}}}+{\text{Δ}}_{h}\right)V_{h}$$

where ${\text{Δ}}_{h}$ is the dynamic bias that adapts attention weights based on input features. The dynamic bias, ${\text{Δ}}_{h}$ is computed as follows,


$${\text{Δ}}_{h}=\text{ReLU }\left(X_{l}W_{d}^{h}+b_{d}^{h}\right)$$

The outputs from all attention heads are concatenated and projected back to the embedding space using the flowing equation,


$$Z_{l}=\text{Concat }\left(A_{1},A_{2},\ldots ,A_{H}\right)W_{o}$$

where $W_{o}\in {\mathbb{R}}^{C\times C}$ is a learnable weight matrix. The detailed pseudo-code can be seen in **Algorithm 1**.

Algorithm 1 **Encoder with patch embedding and dynamic multi-head self-attention (D-MSA).**

**Require**: Input volume 
$X\in {\mathbb{R}}^{H\times W\times D\times S}$
, patch size (H_0_,W_0_,D_0_), embedding dim C, number of heads H
1: Partition X into non-overlapping 3D patches of size *H*
_0_ × *W*
_0_ × *D*
_0_
2: Flatten patches and reshape to sequence: 
$X^{\prime }\in {\mathbb{R}}^{N\times \left(H_{0}W_{0}D_{0}\right)}$
where 
$N=\frac{H}{H_{0}}\cdot \frac{W}{W_{0}}\cdot \frac{D}{D_{0}}$

3: Project patches to embedding space:

$E=X^{\prime }W_{e}+b_{e}$
, where 
$W_{e}\in {\mathbb{R}}^{H_{0}W_{0}D_{0}\times C}$

4: X*
_l_
* ← *E*
5: **for** each Transformer block layer *l* = 1 to *L* **do**
6:  **//D-MSA computation**
7:  **for** each head *h* = 1 to *H* **do**
8:   
$Q^{h}\leftarrow X_{l}W_{q}^{h}$
,   
$K^{h}\leftarrow X_{l}W_{k}^{h}$
,   
$V^{h}\leftarrow X_{l}W_{v}^{h}$

9:   
${\text{Δ}}^{h}\leftarrow \text{ReLU}\left(X_{l}W_{d}^{h}+b_{d}^{h}\right)$

10:   
$A^{h}\leftarrow \text{Softmax}\left(\frac{Q^{h}{\left(K^{h}\right)}^{⊤}}{\sqrt{d_{h}}}+{\text{Δ}}^{h}\right)V^{h}$

11:  **end for**
12:  Concatenate heads: *Z*
_l_ ← Concat(*A*
^1^,*A*
^2^,…,*A*H)Wo
13: X*
_l_
* ← *Z_l_
*
14: **end for**
15: **return** *X_l_
* {Final encoder output feature sequence}

### 3.2 Decoder

The decoder in our network is structured in a U-shaped design, inspired by the architecture of U-Net. Features extracted from each stage of the encoder are transmitted to the corresponding stage in the decoder through skip connections, preserving critical spatial and semantic information. At each decoder stage, these encoder features are combined with the upsampled decoder features using a FM-M. This module merges the input features by concatenating them along the channel dimension, followed by a convolution operation to ensure seamless blending of information from different scales.

After feature fusion, the output is refined through a RM, which is composed of two 3×3×3 convolutional layers with residual connections and an instance normalization layer. The RM enhances feature representation while ensuring efficient gradient flow during backpropagation, making the training process stable and effective.

The final segmentation output is obtained by processing the reconstructed feature maps through a 1×1×1 convolution layer, followed by a sigmoid activation function. This setup ensures precise pixel-wise segmentation of liver and tumor regions. The integration of skip connections, FM-M, and RM allows the decoder to leverage multi-scale information effectively for accurate reconstruction.

#### 3.2.1 Feature mix module

**Figure 2:**
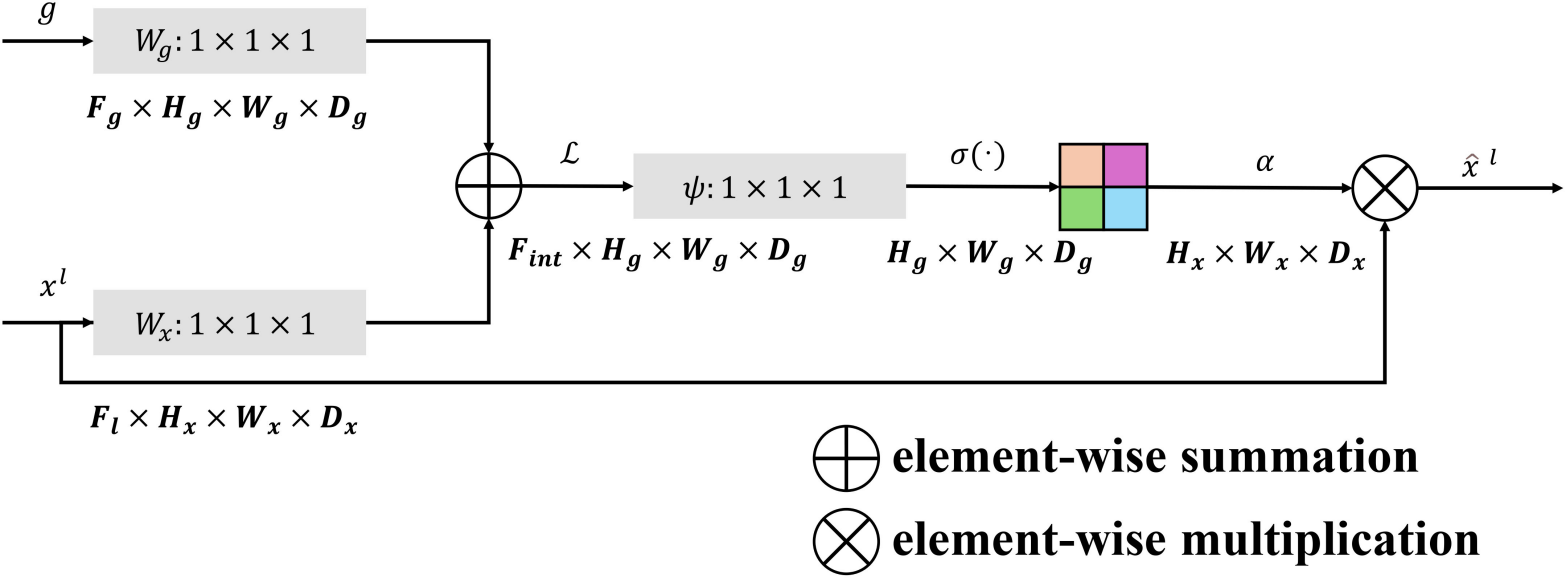
The architecture of the feature mix module which incorporates the multi-scale information.

The FM-M plays a crucial role in merging multi-scale features from the encoder and decoder pathways. By effectively blending spatial and semantic information, this module ensures that the decoder can utilize both high-resolution and context-aware features for accurate segmentation. The FM-M achieves this through a combination of learnable linear projections, attention-guided weighting, and feature fusion. The architecture details of the feature mix module is show in (**Figure 2**).

The module begins by processing the upsampled decoder feature map, $D\in {\mathbb{R}}^{H\times W\times C_{d}}$ , and the corresponding encoder feature map, $E\in {\mathbb{R}}^{H\times W\times C_{e}}$ , where H and W are the spatial dimensions, and $C_{d}$ and $C_{e}$ represent the channel dimensions of the decoder and encoder features, respectively. Both inputs are passed through learnable 1×1×1 convolutional filters, $W_{ɡ}$ and $W_{x}$ , to align their dimensions and emphasize important features:


$$ɡ=W_{ɡ}\cdot D,x=W_{x}\cdot E$$

The attention mechanism then computes an attention map ψ , which dynamically weights the encoder features based on the combined decoder and encoder information. This is achieved through element-wise addition of g and x, followed by a rectified linear unit (ReLU) activation and a sigmoid activation:


$$\psi =\sigma \left(W_{\psi }\cdot \left(\text{ReLU }(ɡ+x\right))\right)$$

where $W_{\psi }$ is another 1×1×1 convolutional filter, and σ denotes the sigmoid activation. The resulting attention map $\psi \in {\mathbb{R}}^{H\times W\times 1}$ acts as a gating mechanism, highlighting the most relevant encoder features for the current decoder stage.

The encoder features are modulated by this attention map through element-wise multiplication:


$$E^{\prime }=\psi \cdot E$$

producing refined encoder features $E^{\prime }$ that are better aligned with the decoder’s requirements. These modulated encoder features are concatenated with the decoder features along the channel dimension:


$$C=\;\text{Concat}\;\left(D,E^{\prime }\right)$$

The concatenated feature map $C\in {\mathbb{R}}^{H\times W\times \left(c_{d}+C_{e}\right)}$ is then forwarded through a 1×1×1 convolutional layer $W_{f}$ , which reduces the dimensionality and integrates the features into a consolidated representation as follows,


$$F=W_{f}\cdot C$$

The output of the FM-M, $F\in {\mathbb{R}}^{H\times W\times C}$ , serves as the refined feature map that feeds into the subsequent layers of the decoder. By incorporating attention mechanisms and weighted feature transformations, the FM-M ensures that the decoder effectively utilizes the most critical spatial and semantic information from both pathways, significantly enhancing the segmentation performance. The relevant pesudo-code for the FM-M module can be seen in **Algorithm 2**.

Algorithm 2 **Feature Mix Module (FM-M).**

**Require:** Decoder feature map 
$D\in {\mathbb{R}}^{H\times W\times C_{d}}$
Encoder feature map 
$D\in {\mathbb{R}}^{H\times W\times C_{e}}$

1: Apply 1 × 1 × 1 convolution to decoder: *g* ← Conv_1×1×1_(D,W_
*g*
_)
2: Apply 1 × 1 × 1 convolution to encoder: x ← Conv_1×1×1_(E,W_x_)
3: Add and apply ReLU: *r* ← ReLU(*g* + *x*)
4: Compute attention map: ψ ← σ(Conv_1×1×1_(r,W*
_ψ_
*))
5: Modulate encoder features: 
$E^{\prime }\leftarrow \psi \cdot E$

6: Concatenate with decoder: *C* ← Concat(*D*, 
$E^{\prime }$
)
7: Fuse features: *F* ← Conv_1×1×1_(*C*,*W_f_
*)
8: **return** *F* {Refined feature for decoder}

#### 3.2.2 Residual module

**Figure 3:**
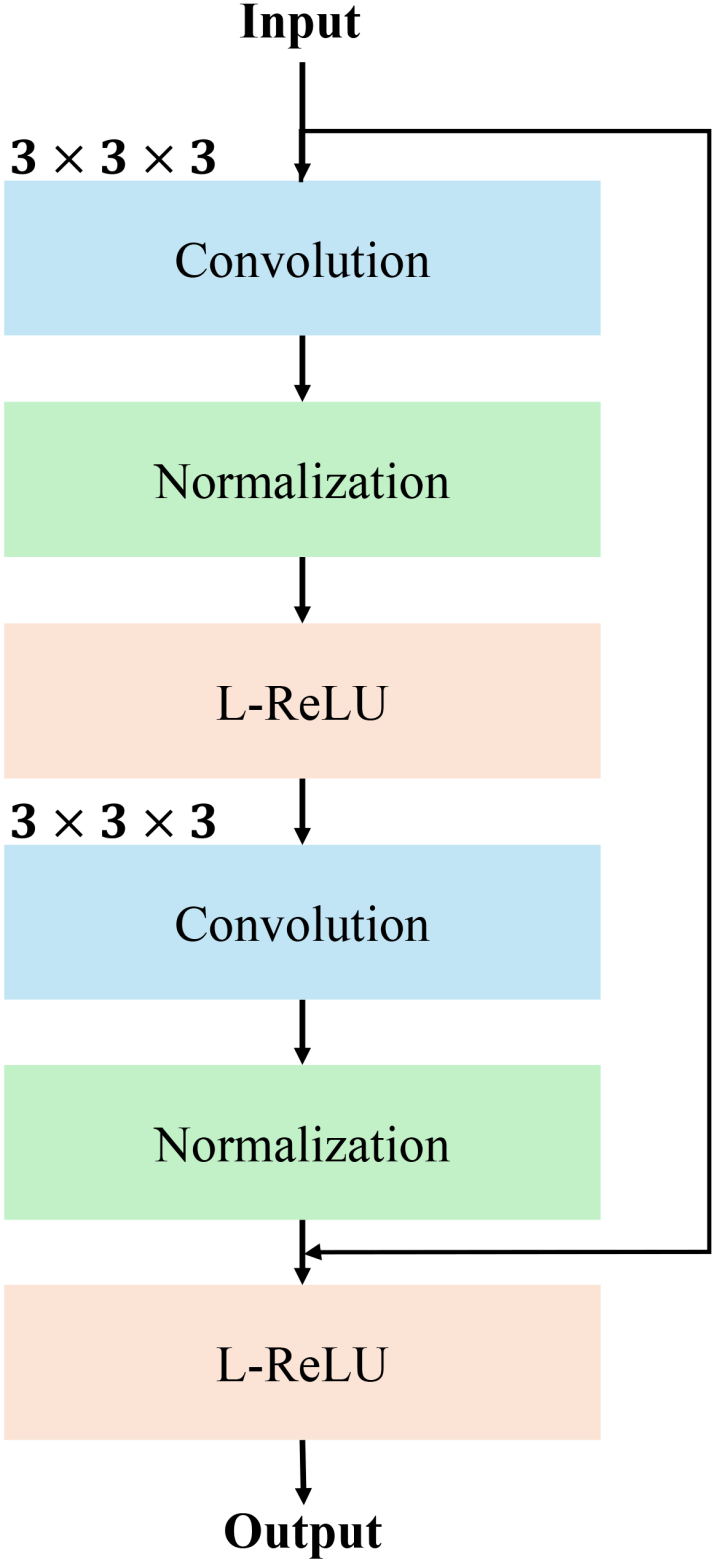
The detailed structure of RM using convolutional normalization and non-linear activation function such as Leaky-ReLU (L-ReLU).

The RM is a crucial component of our network, designed to refine the input feature maps while maintaining efficient gradient flow for stable and effective training. This module follows the principles of residual learning, allowing the model to learn refinements to the input features rather than the features themselves. As illustrated in the figure, the module consists of two consecutive convolutional blocks, each integrated with instance normalization and Leaky ReLU activation, followed by a residual connection to combine the input and processed features. The structure details of RM is show in **Figure 3**.

The input feature map, denoted as $X\in {\mathbb{R}}^{H\times W\times D\times C}$ , where H,W,D are the spatial dimensions and C is the number of channels, is first passed through a 3×3×3 convolution operation. This operation extracts localized spatial features, producing an intermediate feature map:


$$Y_{1}=\text{Conv }3\text{D }\left(X,W_{1}\right)+b_{1}$$

where $W_{1}$ and $b_{1}$ represent the convolution weights and biases. The output is then normalized using instance normalization to stabilize the learning process:


$$Y_{2}=\text{InstanceNorm }\left(Y_{1}\right)$$

Subsequently, a Leaky ReLU (L-ReLU) activation function is applied to introduce non-linearity, defined as:


$$\text{L}-\text{ReLU }(z)=\{\begin{array}{ll} z, & \;\text{if}\;z>0\\ \alpha z, & \;\text{if}\;z\leq 0 \end{array}$$

where *α* is the negative slope parameter, enabling the network to learn more flexible representations.

The processed feature map is subsequently forwarded through a second convolutional block, which applies identical operations: a 3 × 3 × 3 convolution, instance normalization, and Leaky ReLU activation. The output of this block, $Y_{6}$ , represents the refined features. To ensure that the module retains the critical information from the input, a residual connection is added between the original input and the processed features:


$$F=Y_{6}+X$$

This residual connection allows the module to focus on learning residuals to the input features to ensure the original information is preserved while enabling the network to refine and enhance feature representations. The output, F, is a refined feature map of the same dimensions as the input, ${\mathbb{R}}^{H\times W\times D\times C}$ , and is passed to the next layer in the network. The RM’s design ensures robust feature extraction, stable gradient flow, and improved segmentation accuracy. The pesudo-code of RM module is shown in **Algorithm 3**.

Algorithm 3 **Residual module (RM).**

**Require**: Input feature map 
$X\in {\mathbb{R}}^{H\times W\times D\times C}$

1:**//First convolution block**
2: *Y*
_1_ ← Conv_3×3×3_(*X*,*W*
_1_) + *b*
_1_
3: *Y*
_2_ ← InstanceNorm(*Y*
_1_)
4: *Y*
_3_ ← LeakyReLU(*Y*
_2_)
5: **//Second convolution block**
6: *Y*
_4_ ← Conv_3×3×3_(*Y*
_3_,*W*
_2_) + *b*
_2_
7: *Y*
_5_ ← InstanceNorm(*Y*
_4_)
8: *Y*
_6_ ← LeakyReLU(*Y*
_5_)
9:**//Residual connection**
10: *F* ← *X* + *Y*
_6_
11: **return** *F* {Refined output feature map}

## 4 Experimental results analysis

### 4.1 Experimental platform and parameters

Our model implementation was carried out using the PyTorch 2.1.0 deep learning framework, developed in Python 3.8. The experimental environment used an Intel(R) Core(TM) i7-13700KF CPU at 3.40 GHz, paired with an NVIDIA GeForce RTX 4070 Ti SUPER GPU, running on a Windows 10 platform. The hyperparameter details of our proposed model is show in **Table 1**.

**Table 1:** Hyperparameters list of our proposed model.

Parameters	Value
Learning Rate	0.0001
Optimizer	Adam
Loss Function	DiceLoss
Epochs	300
Batch Size	2

### 4.2 Evaluation metrics

The relevant equations for used evaluation metrics is defined as follows,


$$\;\text{Dice}\;\text{Score}\;=\frac{2\sum_{i=1}^{N}p_{i}{ɡ}_{i}}{\sum_{i=1}^{N}p_{i}+\sum_{i=1}^{N}{ɡ}_{i}}$$

$$\;\text{Precision}\;=\frac{\sum_{i=1}^{N}p_{i}{ɡ}_{i}}{\sum_{i=1}^{N}p_{i}}$$

$$\;\text{Recall}\;=\frac{\sum_{i=1}^{N}p_{i}{ɡ}_{i}}{\sum_{i=1}^{N}{ɡ}_{i}}$$

### 4.3 Dataset description

This study utilized two datasets to evaluate the performance of the model such as ATLAS (18) and LiTS (19) datasets. Atlas dataset comprises 90 contrast-enhanced MRI scans focusing on the entire liver in 90 individuals diagnosed with unresectable HCC. Each scan is paired with dedicated segmentation masks delineating both the liver and liver tumors. The Liver Tumor Segmentation (LiTS) dataset comprises 131 training CT scans and 70 additional test scans, acquired from multiple clinical centers via various multi-slice CT scanners. Collectively, these scans encompass many axial slices, each containing expert-delineated labels for both the liver and hepatic tumors. The broad range of imaging protocols and machines ensures rich diversity making LiTS a key resource for testing algorithm generalizability.

For both the ATLAS and LiTS datasets, we applied a structured data preprocessing and augmentation pipeline to enhance consistency and generalization. The pipeline began by loading the images and labels, ensuring the correct channel format, and standardizing spatial resolution and orientation across all volumes. Intensity values were normalized to a fixed range to reduce variability due to differing acquisition settings. Foreground cropping was used to focus on the region of interest, and padding ensured that the spatial dimensions were compatible with the model architecture. To improve the model’s robustness, we employed patch-based sampling with a balanced ratio of foreground and background, followed by a series of spatial and intensity-based augmentations, including random flips, rotations, scaling, and shifting. These transformations helped to simulate real-world variability and reduce overfitting.

### 4.4 Ablation study

We conducted a comparative ablation study using two widely adopted fusion strategies, such as simple concatenation and weighted summation within the DynTransNet framework. As presented in **Table 2**, our FM-M module consistently outperforms the other fusion methods across both the ATLAS and LiTS datasets. Specifically, the proposed module achieves the highest Dice scores of 86.12% (ATLAS) and 93.12% (LiTS), indicating improved segmentation accuracy. Furthermore, it yields notable gains in both precision and recall, demonstrating its ability to retain relevant features while reducing false positives. These improvements highlight the strength of our attention-guided fusion design, which adaptively emphasizes informative features across multiple scales, leading to more precise and reliable segmentation outcomes.

**Table 2:** The ablation study of our proposed FM-M module with simple concatenation and weighted sum method.

Model	Module	Metrics					
		ATLAS			LiTS		
		Dice	Precision	Recall	Dice	Precision	Recall
DynTransNet	FM-M (simple concatenation)	85.74 ± 0.39	86.76 ± 0.37	85.24 ± 0.42	92.85 ± 0.38	94.38 ± 0.36	91.88 ± 0.35
	FM-M (weighted sum)	85.91 ± 0.38	87.21 ± 0.38	85.59 ± 0.38	92.97 ± 0.36	94.53 ± 0.34	91.89 ± 0.38
	FM-M (proposed)	86.12 ± 0.36	87.56 ± 0.31	85.97 ± 0.39	93.12 ± 0.35	94.78 ± 0.33	92.25 ± 0.37

**Table 3** illustrates the ablation study of our proposed model on the ATLAS and LiTS datasets, evaluated using Dice score, Precision, and Recall. To enhance the statistical reliability of our results, we also report the mean ± standard deviation (SD), 95% confidence intervals (CI), and p-values obtained from paired t-tests against the baseline. The baseline model achieved a Dice score of 84.16 ± 0.53 (CI: 83.83–84.49) on ATLAS and 90.58 ± 0.59 (CI: 90.21–90.95) on LiTS. Adding D-MSA to the baseline led to statistically significant improvements (p ¡ 0.05), increasing the Dice score to 84.99 ± 0.57 (ATLAS) and 91.64 ± 0.74 (LiTS). The inclusion of both D-MSA and FM-M yielded further gains, with Dice scores reaching 85.79 ± 0.42 (CI: 85.56–86.02) on ATLAS and 92.49 ± 0.45 (CI: 92.24–92.75) on LiTS. The best performance was observed when all three submodules—D-MSA, FM-M, and RM—were combined, achieving Dice scores of 86.12 ± 0.36 (CI: 85.90–86.34) on ATLAS and 93.12 ± 0.35 (CI: 92.92–93.32) on LiTS, with highly significant p-values (p< 0.001). These results clearly demonstrate that each proposed submodule contributes positively to segmentation performance and that the full model achieves the most accurate and robust results across both datasets.

**Table 3:** The ablation study of our proposed model to evaluate the performance of submodules.

Module	Baseline	Submodules			Metrics							
					ATLAS				LiTS			
		D-MSA	FM-M	RM	Dice (± SD 95% CI)	P-value	Precision	Recall	Dice (± SD 95% CI)	P-value	Precision	Recall
DynTransNet	✓	–	–	–	84.16 ± 0.53 (83.83-84.49)	–	84.47 ± 0.63	83.11 ± 0.65	90.58 ± 0.59 (90.21-90.95)	–	91.67 ± 0.71	89.83 ± 0.64
	✓	✓	–	–	84.99 ± 0.57 (84.64-85.34)	0.0102	85.83 ± 0.64	83.93 ± 0.69	91.64 ± 0.74 (91.18-92.10)	0.0008	92.78 ± 0.81	90.77 ± 0.77
	✓	–	✓	–	84.57 ± 0.76 (84.10-85.04)	0.6912	85.62 ± 0.89	83.79 ± 0.68	91.82 ± 0.61 (91.44-92.20)	0.1233	92.94 ± 0.73	90.92 ± 0.69
	✓	–	–	✓	84.64 ± 0.82 (84.13-85.15)	0.9473	85.71 ± 0.78	84.76 ± 0.71	91.29 ± 0.85 (90.76-91.82)	0.0004	92.36 ± 0.64	90.41 ± 0.72
	✓	✓	✓	–	85.79 ± 0.42 (85.53-86.05)	0.0007	86.95 ± 0.58	84.89 ± 0.49	92.49 ± 0.43 (92.21-92.77)	0.0005	93.51 ± 0.46	91.55 ± 0.45
	✓	✓	–	✓	85.42 ± 0.49 (85.12-85.72)	0.0113	86.34 ± 0.52	84.48 ± 0.47	92.46 ± 0.51 (92.14-92.78)	0.0003	93.38 ± 0.47	91.48 ± 0.56
	✓	–	✓	✓	85.56 ± 0.48 (85.26-85.86)	0.0127	86.59 ± 0.51	84.61 ± 0.54	92.37 ± 0.43 (92.07-92.67)	0.0006	93.16 ± 0.41	91.20 ± 0.53
	✓	✓	✓	✓	86.12 ± 0.36 (85.90-86.34)	0.0001	87.56 ± 0.31	85.97 ± 0.39	93.12 ± 0.35 (92.92-93.32)	0.0001	94.78 ± 0.33	92.25 ± 0.37

### 4.5 Quantitative analysis

**Figure 4:**
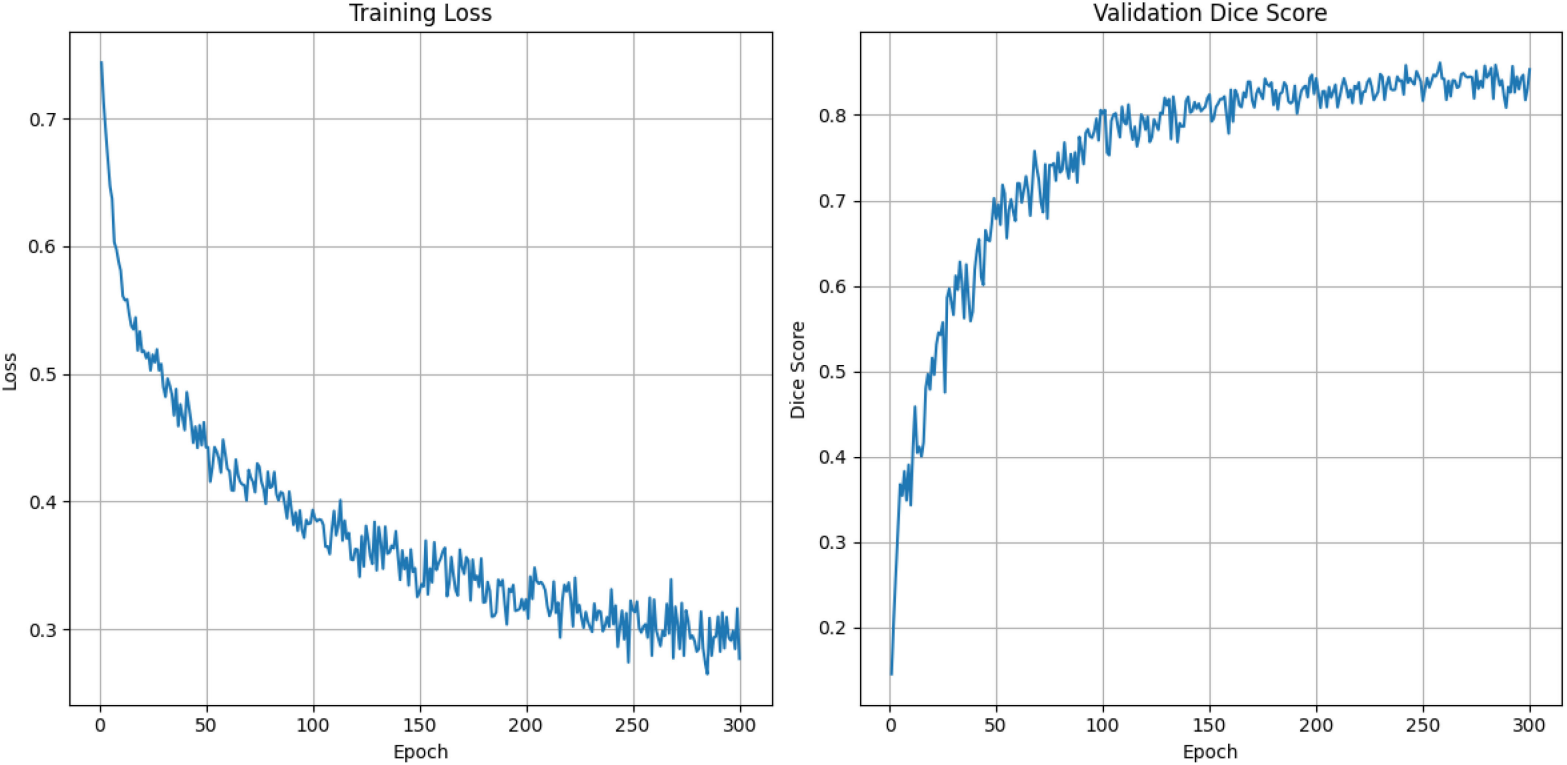
Training loss and validation dice score graph for 300 epochs using DynTransNet on ATLAS dataset.

**Figure 4** represents the training dynamics of our model over 300 epochs in terms of training loss and validation Dice score. The training loss graph indicates a steady decrease as the number of epochs increases. Starting at approximately 0.7, the loss significantly reduces during the initial epochs, reflecting effective model learning. Beyond 150 epochs, the loss converges around 0.3, with minimal fluctuations. The validation Dice score graph demonstrates consistent improvement over the epoch. Initially, the Dice score starts near 0 and rises rapidly over the epochs eventually stabilizing around a Dice score of 0.86. Notably, the model achieved a mean Dice score of 86.12, which indicates our model’s ability to generalize in our validation data.

**Table 4** shows the segmentation performance across multiple models using three key metrics: Dice score, Precision, and Recall. Among the models, SegResNet demonstrates strong performance with a Dice score of 85.31, a Precision of 86.67, and a Recall of 84.50. SwinUNETR and DynUNet also achieved competitive results, with Dice scores of 83.23 and 82.67, respectively. Other models, such as UNETR and VNet achieved slightly lower Dice scores of 80.19 and 81.59 and ViTAutoEncoder lags significantly, achieving a Dice score of 59.85. On the other hand, our proposed model achieves the best results across all metrics, with a Dice score of 86.12, a Precision of 87.52, and a Recall of 85.82. These findings emphasize the capability of our method to achieve accurate and consistent segmentation of the target regions, outperforming current state-of-the-art techniques. From a clinical perspective, even marginal gains in Dice score can significantly improve tumor localization and treatment planning by reducing variability and enhancing boundary precision. The higher overlap between predicted and actual tumor regions supports more reliable radiological assessment and intervention decisions.

**Table 4:** Comparison of different models based on dice score, precision, and recall.

Model name	Dice score	Precision	Recall
SegResNet (20)	85.31	86.67	84.50
DynUNet (21)	82.67	83.26	81.04
UNETR (22)	80.19	81.21	79.35
SwinUNETR (23)	83.23	84.46	82.78
ViTAutoEnc (24)	59.85	60.78	58.51
VNet (25)	81.59	82.49	80.66
DynTransNet	86.12	87.52	85.82

**Figure 5:**
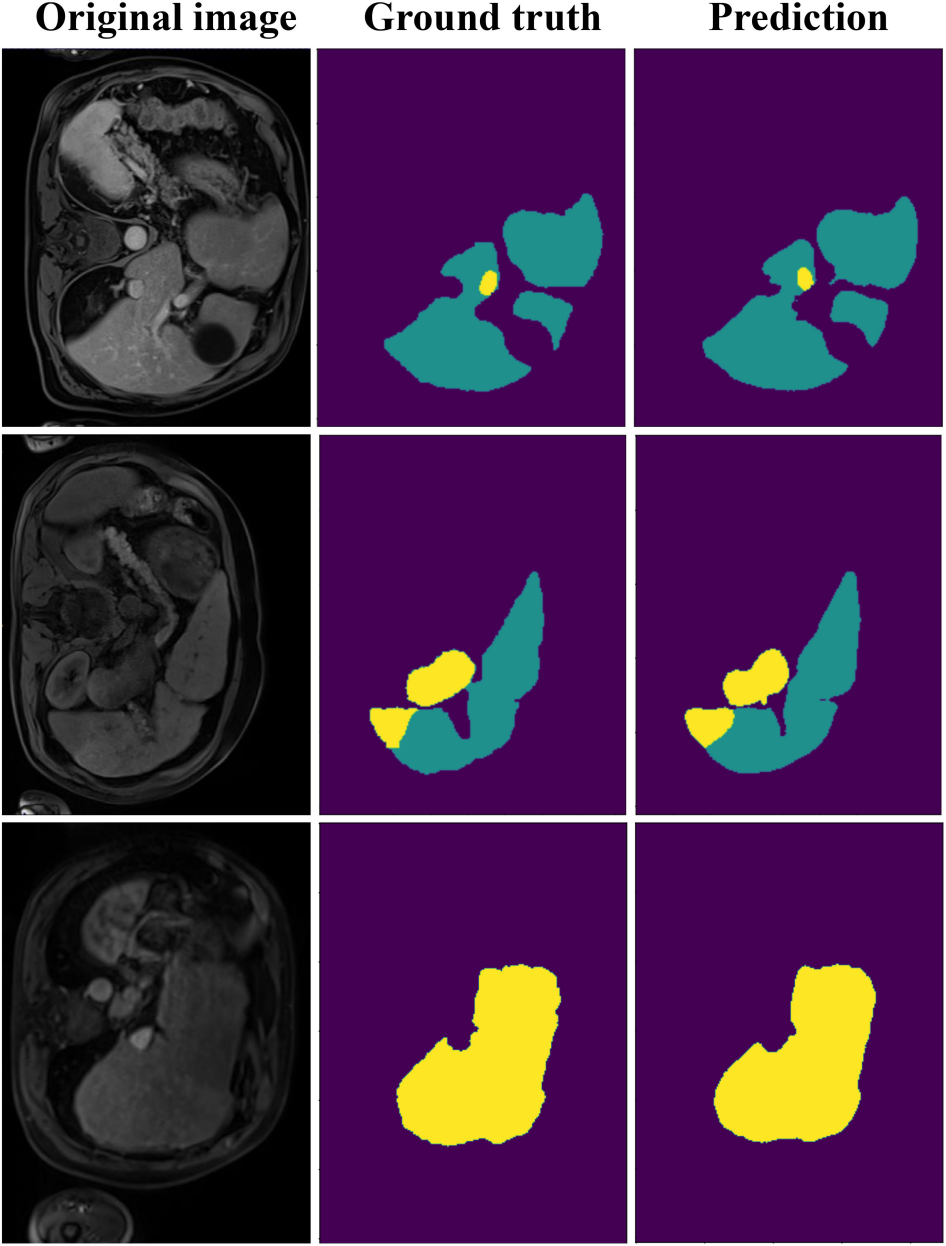
The visualization result of DynTransNet using ATLAS dataset.

**Figure 5** illustrates liver and tumor segmentation results, comparing ground truth annotations with the model’s predictions across three different slices of abdominal imaging. The ground truth shows the accurate delineation of the liver and tumor regions, marked in distinct colors. The predictions closely align with the ground truth, demonstrating our model’s ability to accurately segment both the liver and tumors. While minor deviations may exist at some boundaries, the overall segmentation quality highlights the robustness and precision of the proposed model, effectively capturing both large liver structures and smaller, irregularly shaped tumor regions.

**Figure 6:**
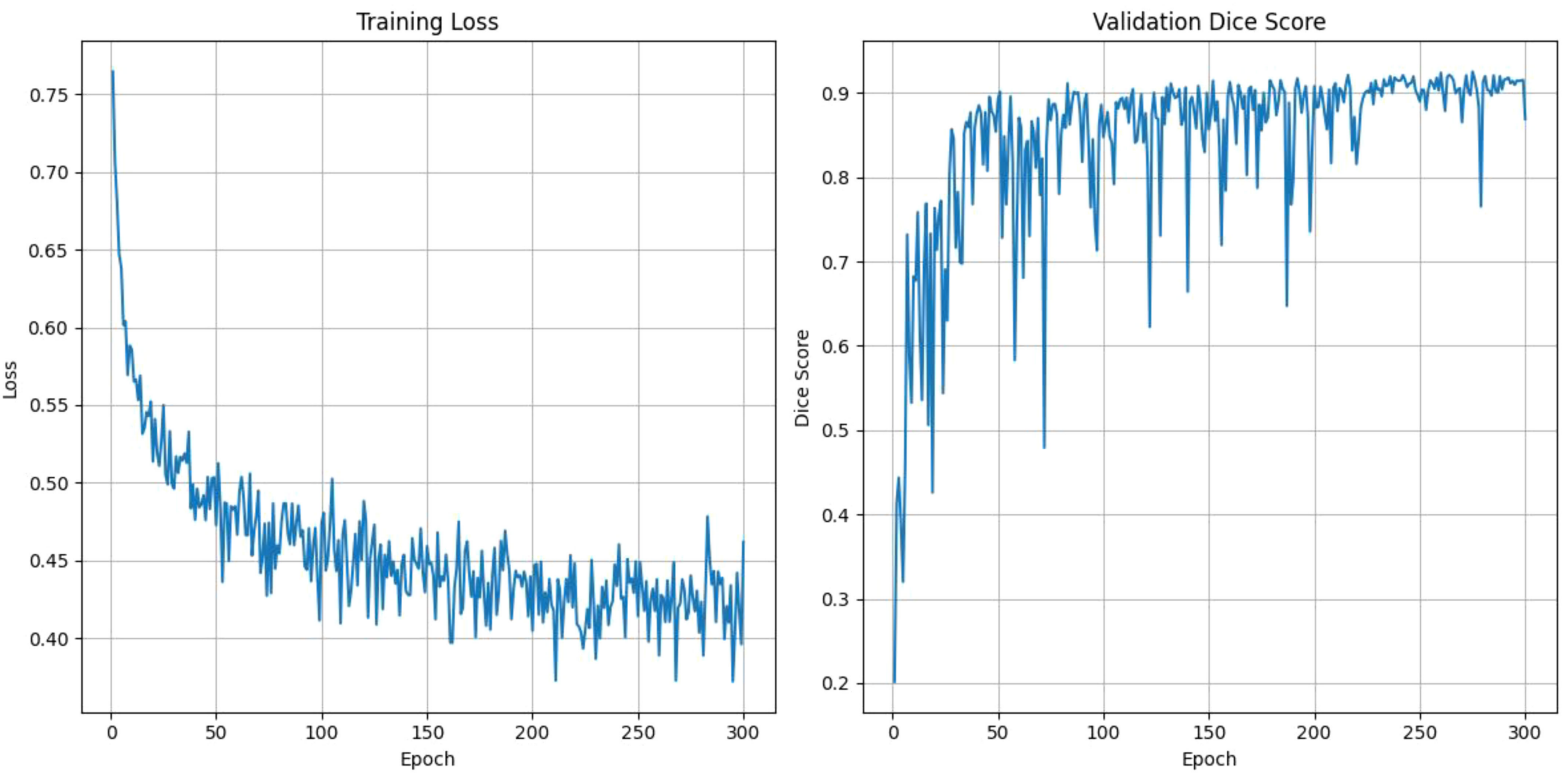
The training loss and validation dice score over the epochs for DynTransNet using LiTS dataset.

**Figure 6** shows the training loss and validation Dice score over 300 epochs. The training loss shows a steady decline, starting at around 0.75 and gradually reducing to approximately 0.4, which shows consistent improvement in the model’s learning. Meanwhile, the validation Dice score rapidly increases during the initial epochs, reaching to above 0.9 by around 50 epochs and maintaining stability with minor fluctuations throughout the remaining epochs. This trend highlights the model’s ability to generalize well and high segmentation accuracy while avoiding overfitting as the training progresses.

**Table 5** shows the performance comparison on the LiTS dataset for liver and tumor segmentation. DeepLabv3+ leads in Precision with 96.10, while HiFormer achieves the highest Recall at 93.71. In contrast, LightM-UNet records the lowest Dice score at 84.58. Our model performs consistently well across all metrics, achieving a Dice score of 93.12, Precision of 94.05, and Recall of 92.25, which demonstrates our model’s robustness in segmenting both liver and tumor regions. This high Dice score reflects a strong spatial overlap between predicted and ground truth annotations, which is clinically significant for accurate tumor boundary identification. Such precision supports more effective surgical planning and radiation targeting, potentially leading to improved treatment outcomes and reduced procedural risks.

**Table 5:** Comparison of different models based on dice score, precision, and recall.

Model name	Dice score	Precision	Recall
U-Net (26)	87.19	87.24	90.82
Attention U-Net (27)	89.75	92.91	90.15
DeepLabv3+ (28)	92.95	96.10	92.14
TransUNet (29)	86.71	89.49	87.89
HiFormer (30)	93.06	94.60	93.71
G-Gascade (31)	93.01	95.49	92.87
LightM-UNet (32)	84.58	–	–
DynTransNet	93.12	94.78	92.25

**Figure 7:**
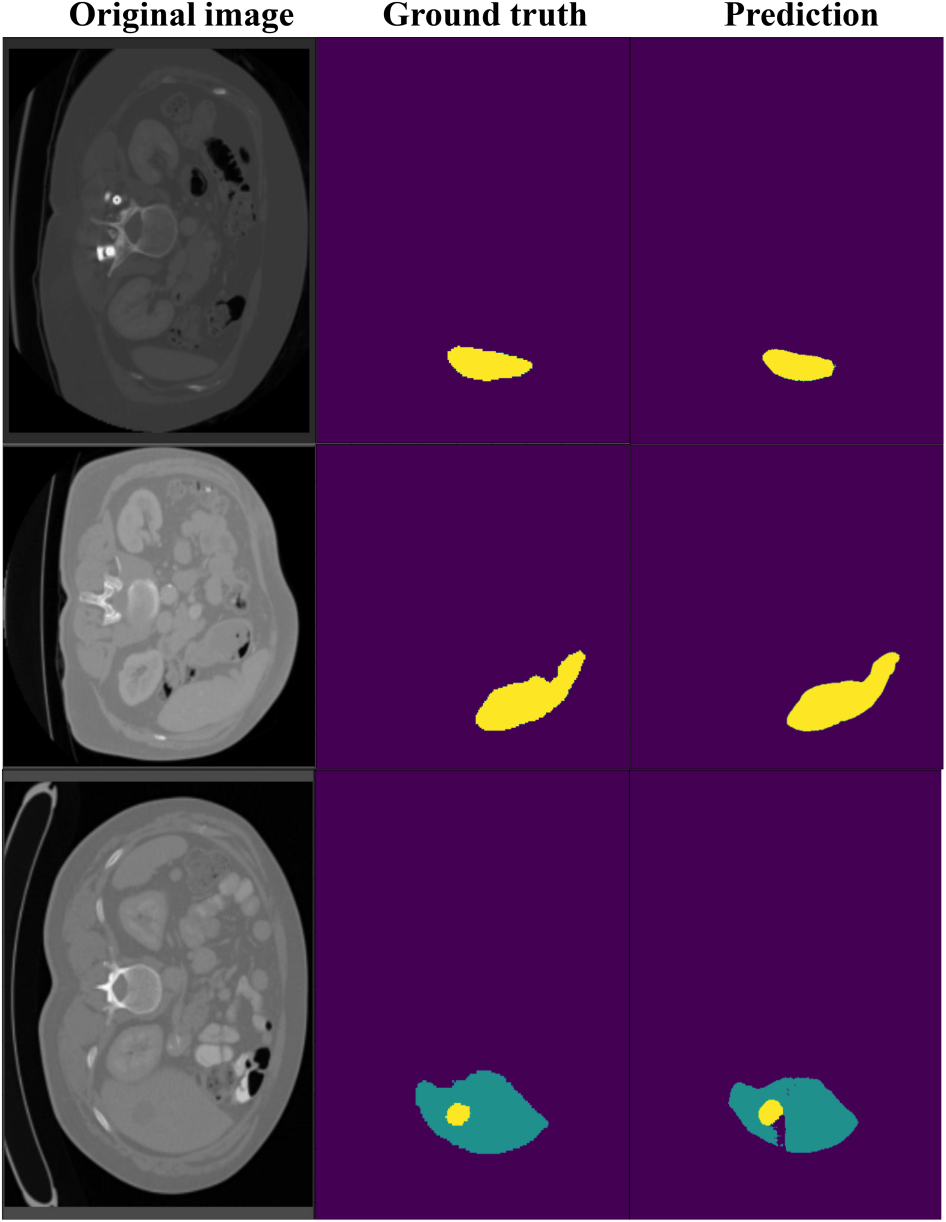
The visualization of DynTransNet on LiTS dataset to evaluate the performance of the model.

**Figure 7** shows the segmentation results on the LiTS dataset, comparing ground truth and predicted segmentations for liver and tumor regions across three CT slices. The ground truth annotations provide accurate delineations of liver and tumor areas, while the model predictions closely match these annotations, demonstrating high segmentation accuracy. Notably, even in small or irregularly shaped tumors, the model successfully captures their boundaries with minimal deviations from the ground truth. These results highlight our model’s effectiveness in handling challenging cases and its reliability for clinical applications.

## 5 Conclusion

This work proposes DynTransNet, a deep learning architecture designed for accurate liver and tumor segmentation in multi-modal medical imaging (CT and MRI). Built on a U-shaped backbone, the framework incorporates Dynamic Multi-Head Self-Attention and a Feature Mix Module to enhance spatial dependency modeling and hierarchical feature fusion. Evaluated on the ATLAS and LiTS datasets, DynTransNet achieved Dice scores of 86.12 and 93.12, respectively, outperforming current state-of-the-art methods. Beyond quantitative performance, these results indicate the model’s strong potential for supporting radiologists in clinical tasks by reducing manual segmentation time, enhancing consistency, and improving tumor localization.

However, the current study has several limitations. The model was trained and evaluated on publicly available datasets, which may not fully capture the variability present in real-world clinical settings. Additionally, the computational complexity of the architecture may pose challenges for deployment in resource-constrained environments.

In future work, we aim to explore real-time deployment, reduce computational overhead for integration into clinical workflows, and validate the framework across larger, multi-institutional datasets to ensure robustness and generalizability in real-world medical environments.

## Data Availability

The original contributions presented in the study are included in the article/supplementary material. Further inquiries can be directed to the corresponding author.
